# The impact of COVID-19 epidemic on emergency department visits of older patients in Taiwan

**DOI:** 10.1186/s12877-023-04164-x

**Published:** 2023-08-14

**Authors:** Hao-Ming Chang, Chih-Chen Chang, Pei-Ying Lin, Yi-Chen Lee, Hsien-Hao Huang, David Hung-Tsang Yen

**Affiliations:** 1 Department of Emergency Medicine, Taipei Veterans General Hospital, No. 201, Section 2, Shih-Pai Road, Taipei, 112 Taiwan; 2 Department of Emergency Medicine, Taoyuan Branch, Taipei Veterans General Hospital, Taoyuan, 330 Taiwan; 3 Faculty of Medicine, National Yang Ming Chiao Tung University, Taipei, 112 Taiwan; 4 Institute of Emergency and Critical Medicine, School of Medicine, National Yang Ming Chiao Tung University, Taipei, 112 Taiwan; 5 Chang Bing Show-Chwan Memorial Hospital, Changhua, 505 Taiwan; 6 Department of Nursing, Yuanpei University of Medical Technology, Hsinchu, 300 Taiwan

**Keywords:** Older patients, Emergency department, COVID-19 infection

## Abstract

**Background:**

The number of emergency department (ED) visits has significantly declined since the COVID-19 pandemic. In Taiwan, an aged society, it is unknown whether older adults are accessing emergency care during the COVID-19 epidemic. Therefore, this study aimed to investigate the impact of COVID-19 on the ED visits and triage, admission, and intensive care unit (ICU) hospitalization of the geriatric population in a COVID-19-dedicated medical center throughout various periods of the epidemic.

**Methods:**

A retrospective chart review of ED medical records from April 9 to August 31, 2021 were conducted, and demographic information was obtained from the hospital’s computer database. The period was divided into pre-, early-, peak-, late-, and post-epidemic stages. For statistical analysis, one-way analysis of variance followed by multiple comparison tests (Bonferroni correction) were used.

**Results:**

A statistically significant decrease in the total number of patients attending the ED was noted during the peak-, late-, and post-epidemic stages. In the post-epidemic stage, the number of older patients visiting ED was nearly to that of the pre-epidemic stage, indicating that older adults tend to seek care at the ED earlier than the general population. Throughout the entire epidemic period, there was no statistically significant reduction in the number of the triage 1& 2 patients seeking medical attention at the emergency department. In the entire duration of the epidemic, there was no observed reduction in the admission of elderly patients to our hospital or ICU through the ED. However, a statistically significant decrease was observed in the admission of the general population during the peak epidemic stage.

**Conclusions:**

During the peak of COVID-19 outbreak, the number of ED visits was significantly affected. However, it is noteworthy that as the epidemic was gradually controlled, the older patients resumed their ED visits earlier that the general population as indicated by the surge in their number. Additionally, in the patient group of triage 1& 2, which represents a true emergency, the number did not show a drastic change.

## Background

Coronavirus disease 2019 (COVID-19) was first reported in Wuhan, China [1]. Since then, it has spread to multiple countries within months [2, 3]. On March 11, 2020, the World Health Organization officially declared the COVID-19 outbreak a global pandemic [4]. To date, this novel virus has caused more than 760 million confirmed cases and 6.8 million deaths worldwide.

As an island nation, Taiwan has an inherent advantage when it comes to border control management [5]. Methods including real-time surveillance with rapid risk assessment, laboratory capacity building, extensive case detection, rigorous contact tracing, and daily monitored mandated quarantines with a people-centered approach have successfully curbed community spread, while schools and businesses remain open [6–8]. In Taiwan, since the start of the COVID-19 pandemic, there have been only few sporadic COVID-19 confirmed cases in the first half of 2020; thus, it has been considered highly successful in the initial rapid containment of the COVID-19 pandemic [9]. There were even 222 consecutive days without a locally transmitted case from April 12 to November 21, 2020 [10]. However, sporadic cases of COVID-19 began to appear in late April, 2021. The first case was a cargo flight pilot from Indonesia who had contracted the virus with only mild symptoms of an itchy throat after flying to Australia on April 20, 2021. The Central Epidemic Command Center (CECC) immediately started an investigating the pilot’s history of activity in Taiwan and identified individuals who had come into contact with him. Of the identified cases, two tested positive and their COVID-19 diagnosis was confirmed on April 23, 2021. The CECC subsequently launched an investigation to look for further potential indigenous cases and implemented a “Clearing Plan” for China Airlines. Unfortunately, this outbreak escalated into the largest COVID-19 outbreak in Taiwan since the start of the epidemic, with seven more indigenous cases reported on May 11, 2021, six of which had no known sources of infection. To prevent wider community transmission, the CECC raised the epidemic warning to level 2 and implemented several related restrictions and measures on the same day.

Generally, regional infectious disease outbreaks or natural disasters are associated with noticeable changes in the number of emergency department (ED) visits, especially for non-COVID-19 visits [11, 12]. In 2003, severe acute respiratory syndrome (SARS) outbreak affected the medical service system and it might be associated with a 40% reduction in visits to the ED [13]. Similar to that of SARS outbreak, people became reluctant to hospitals due to panic and fear of contracting COVID-19 [14, 15]. Relative to the pre-pandemic period, the delay in treatment from symptom onset to hospital arrival is common during a pandemic, resulting in worse prognoses and more urgent clinical conditions of patients; thus, ultimately increasing morbidity and mortality associated with both chronic and acute health conditions [16]. Conversely, the CECC introduced prevention programs that could help decrease the spread of communicable disease, including wearing the surgical mask at all times while outside, practicing appropriate social distancing at restaurants and department stores, and washing hands regularly [17, 18].

Recent studies have shown a significant reduction in ED visits over the first weeks of the pandemic [11, 19]. The reasons might be that either patients avoided hospital visits due to fear of contracting COVID-19 or transmission of certain communicable diseases had slowed because of fewer human contact [14, 17, 20]. However, these studies did not assess changes in clinical characteristics, emergency status, and admission rate among ED attendees. Additionally, they only investigated the early phase of pandemic and during lockdown, without assessing the change in trends during the different types of lockdown. Moreover, Taiwan had more than 7% of its population aged ≥ 65 years, becoming an “aging society” in 1993 and the proportion exceeded 14% in 2006, so Taiwan officially entered a new era, an “aged society” [21]. The proportion has already exceeded 20% in 2018. The rapid increase in the proportion of elderly people is a situation that we must take it seriously. Taipei Veterans General Hospital (TVGH) mainly serves older patients. The impact of the COVID-19 epidemic on the geriatric population needing emergency services was not fully recognized.

This study aimed to investigate the impact of COVID-19 on ED visits and triage, admission, and intensive care unit (ICU) hospitalization of the geriatric population in a COVID-19-dedicated medical center throughout various periods of the epidemic.

## Methods

### COVID-19 epidemiology

TVGH is a tertiary referral and teaching medical center in northern Taiwan with 2,700 beds. From 2018 to 2020, the ED of the hospital handled an average of 80,791 visits yearly. There have been only a few sporadic outbreaks in Taiwan since the COVID-19 pandemic began. TVGH, one of the designated COVID-19 hospitals in Taiwan, was established for identifying potential patients with COVD-19 in the ED and had more than 100 isolation beds for COVID-19 patient care during the outbreak period in mid-2021. Data on all patients including the number of ED visits, age, services provided, triage categories, and admission to ward and ICU were retrieved from the hospital computer database for analysis.

### Study population and protocol

We defined the pre-epidemic stage as the month prior to May 9, 2021 and the early epidemic stage as period from May 10 to May 18, due to May 10 being the day when CECC confirmed the presence of several indigenous cases and suspected the community spread. The peak-epidemic stage was the period from May 19 to June 24, when CECC raised epidemic warnings to Level 3 nationwide. Since June 25, the CECC daily confirmed indigenous COVID-19 cases down to 76 patients and continuously decreased. The period from June 25 to July 26 was defined as the late-epidemic stage. The post-epidemic stage was from July 27 to August 31, the time when the CECC announced a lower epidemic alert level of Level 2 and a similar time interval as the pre-epidemic stage.

A retrospective chart review of ED medical records was conducted and demographic information was obtained from the hospital’s computer database. Patient data were collected on alternate days throughout the study duration and 23,691 patients were recruited. Patient information that was reviewed, analyzed, and compared for different stages of the COVID-19 epidemic with the general and older groups (aged ≥ 65 years) included the total number of visits, age, triage category, ED visit for trauma and non-trauma, and status after the ED visit. The triage category was defined as follows: category 1: true emergency, life-threatening if not treated immediately; category 2: emergency, moderate abnormal vital signs; category 3: urgent, emergency condition but not serious enough for category 2; category 4: non-urgent, not an emergency condition or possibly an acute condition without the need for laboratory tests or minor symptoms with an obvious diagnosis; and category 5: not urgent. The number of daily laboratory-confirmed COVID-19 cases was collected from the Taiwan Centers for Disease Control.

### Statistical analysis

Data were presented as mean ± standard deviation or mean with 95% confidence interval (CIs). SPSS version 10.0 for Windows (SPSS Inc., Chicago, IL, USA) was used for data analysis. One-way ANOVA was used for statistical analysis, followed by multiple comparison tests (Bonferroni correction). There was total 35 comparisons in our figure, so a *P*-value of < 0.0014 (0.05/35) was considered statistically significant.

## Results

**Fig. 1:**
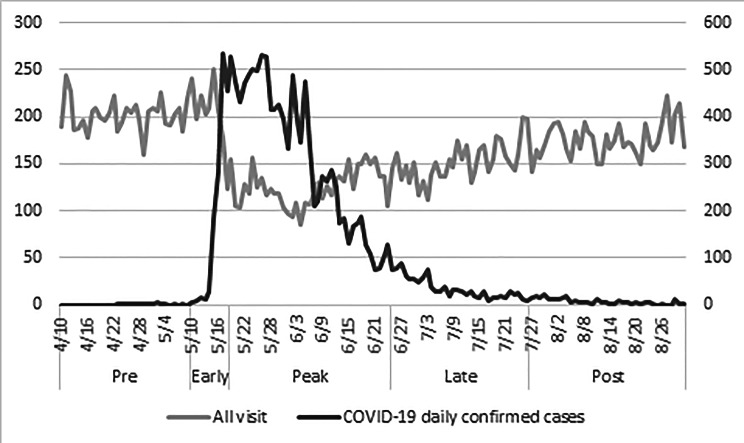
Daily ED visits and number of COVI-19 daily confirmed cases. Total number of emergency department visits and number of COVID-19 daily confirmed cases in the pre-epidemic, early-epidemic, peak-epidemic, late-epidemic, and post-epidemic stages during local transmission in Taiwan between April and August 2021

**Fig. 2:**
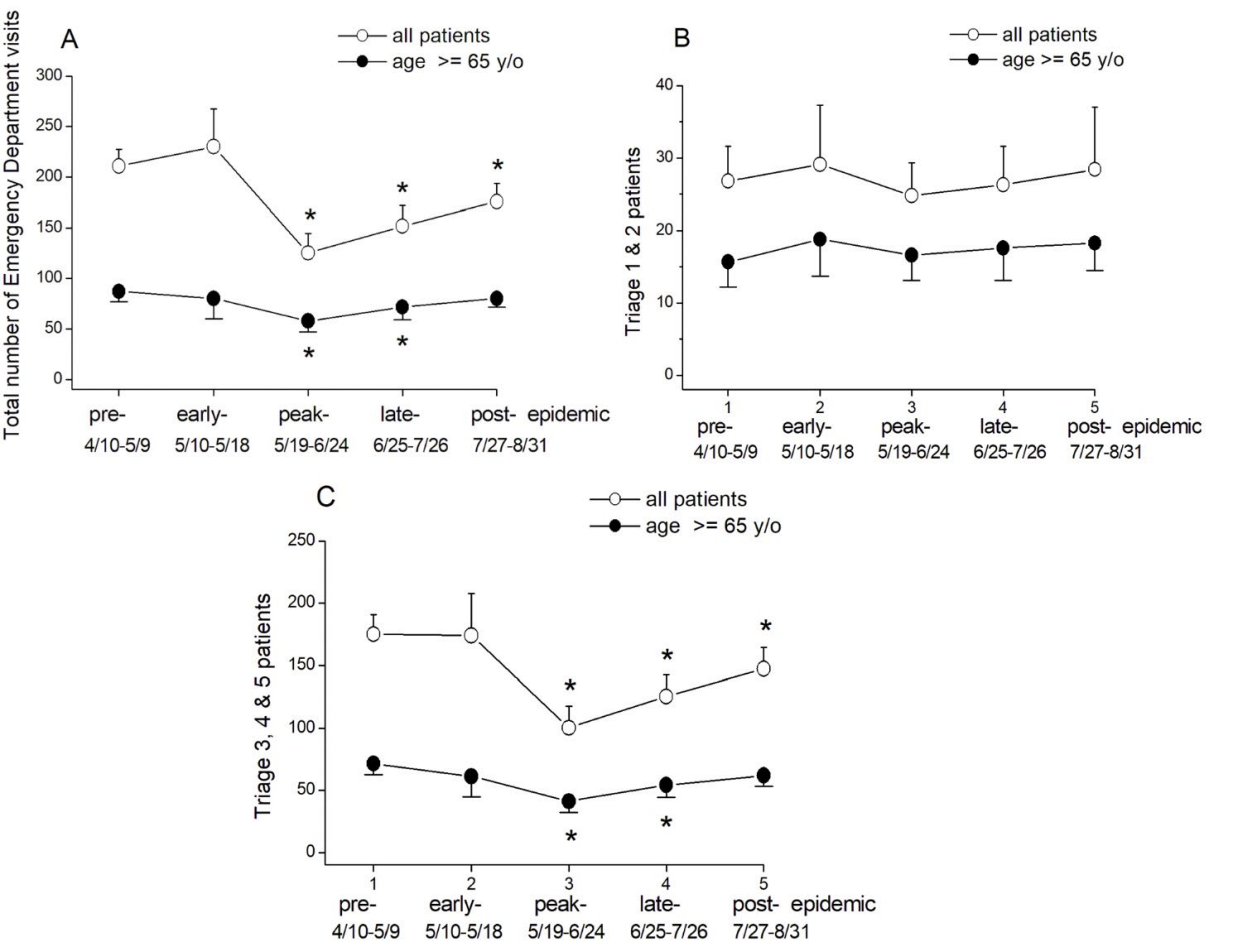
Comparison by emergency department visits and patient triage category. Comparison between all patients and patients aged ≥ 65 years in emergency department visits (**A**), patient triage category 1&2 (**B**) and 3, 4&5 (**C**) in the pre-epidemic, early-epidemic, peak-epidemic, late-epidemic, and post-epidemic stages during local transmission in Taiwan between April and August 2021. (Values are presented as mean ± standard deviation. *p < 0.0014, compared with the pre-epidemic stage)

In 2021, from January to August, the ED handled a total of 43,272 visits, an 8.5% (n = 47,298) and 25% (n = 57,730) decrease from 2020 to 2019, respectively. During the early-epidemic stage, from May 10 to May 18, there was a marked surge in the number of confirmed COVID-19 cases (Fig. 1). Most of these cases are concentrated in northern Taiwan, and a significant number of these cases were treated at TVGH, a medical center located in Taipei City. The average daily ED visits in the TVGH during the different stages of epidemic are shown in Fig. 2A. During the pre-epidemic and early-epidemic stages, the total number of patients visiting the ED remained relatively stable (201.8 ± 16.7 and 203.2 ± 37.3, respectively). On May 19, the CECC raised the epidemic warnings to Level 3 nationwide. The total number of patients visiting the ED daily significantly decreased to 125.2 ± 19.4 (p < 0.001) in the peak epidemic stage, 151.7 ± 20.4 (p < 0.001) in late-epidemic stage, and 175.7 ± 18.2 (p < 0.001) in post-epidemic stage, as compared with that in pre-epidemic stage. However, we observed a significant decrease in the number of elderly patients visiting the ED in peak-epidemic stage (57.9 ± 10.6, p < 0.001), and late-epidemic stage (71.7 ± 12.6, p < 0.001), respectively, but had no significant differences in post-epidemic stage (80.2 ± 8.6, p > 0.0014), as compared with the pre-epidemic period (87.2 ± 10.0) (Fig. 2A). Additionally, we found that elderly patients showed an earlier increase in ER visits in the post-epidemic stage compared to the general population. These findings suggest a higher demand for emergency services among elderly patients compared to the general population.

The triages 1 and 2 (Fig. 2B), which indicated that the patient was in a true emergency or in an emergency with abnormal vital signs, noted no significantly difference in patient number during the COVID-19 epidemic, in both the general (26.8 ± 4.8, 29.1 ± 8.2, 24.8 ± 4.5, 26.3 ± 5.3, and 28.4 ± 8.6) and older population (15.7 ± 3.5, 18.8 ± 5.1, 16.6 ± 3.5, 17.6 ± 4.5, and 18.3 ± 3.8). However, for the triages 3, 4, and 5 (Fig. 2C), which correspond to less severe conditions, there was a statistically significant decrease in patient numbers during the peak-, late-, and post-epidemic stage, in the general population (100.3 ± 17.3, 125.3 ± 17.3, 147.3 ± 17.1, respectively), compared to the pre-epidemic stage (175.0 ± 15.9). In the older population, there was also a statistically significant decrease during the peak- and late-epidemic stage (41.2 ± 9.1, 54.2 ± 9.9, respectively), but in the post-epidemic stage, there was no significant differences (61.9 ± 7.6, p > 0.0014), compared to the pre-epidemic stage (71.5 ± 8.8).

**Fig. 3:**
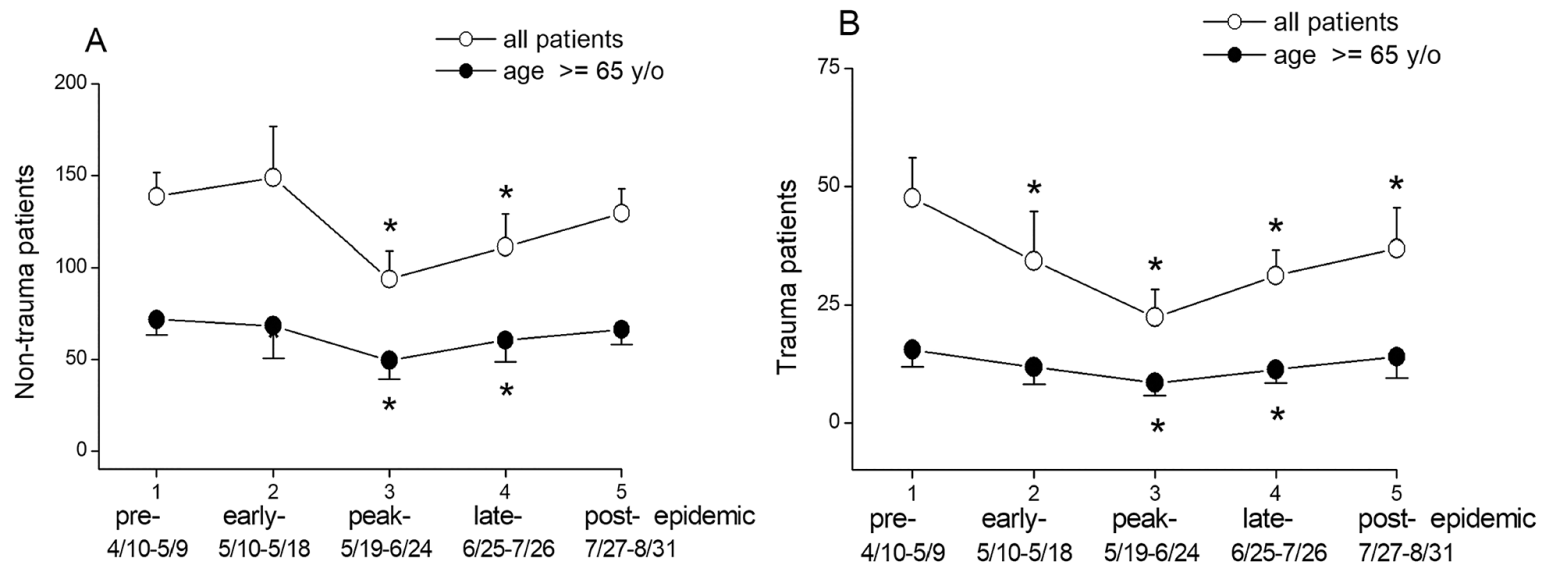
Comparison by non-trauma and trauma category. Comparison between all patients and patients aged ≥ 65 years in non-trauma (**A**) and trauma (**B**) category in the pre-epidemic, early-epidemic, peak-epidemic, late-epidemic, and post-epidemic stages during local transmission in Taiwan between April and August 2021. (Values are presented as mean ± standard deviation. *p < 0.0014, compared with the pre-epidemic stage)

In non-trauma cases (Fig. 3A), both the total number of patients and older patients showed a significantly decrease during the peak- (93.8 ± 15.1, p < 0.001 and 49.4 ± 10.1, p < 0.001) and late-epidemic stages (111.2 ± 17.7, p < 0.001 and 60.4 ± 11.7, p = 0.001, respectively), compared to the pre-epidemic stage (138.5 ± 12.9, and 71.7 ± 8.5). However, in the post-epidemic stage, there were no significant differences in the total number of patients and older patients visiting the ED compared to the pre-epidemic stage.

In trauma cases (Fig. 3B), there was a statistically significant reduction in the total number of patients during the early-, peak-, late-, and post-epidemic stages (34.3 ± 10.4, 22.4 ± 5.8, 31.2 ± 5.4, and 36.9 ± 8.6, all p < 0.001, respectively) compared to the pre-epidemic stage (47.5 ± 8.6). Among elderly patients, there was a statistically significant reduction during the peak-epidemic and post-epidemic stages (8.5 ± 2.7 and 11.3 ± 2.9, both p < 0.001, respectively), while no significant reduction was observed in the early-epidemic and post-epidemic stages (11.8 ± 3.6 and 14.0 ± 4.5, respectively), compared to the pre-epidemic stage (15.5 ± 3.6). These findings suggested that the ED visits of elderly trauma patients were not affected during early- and post-epidemic stage.

**Fig. 4:**
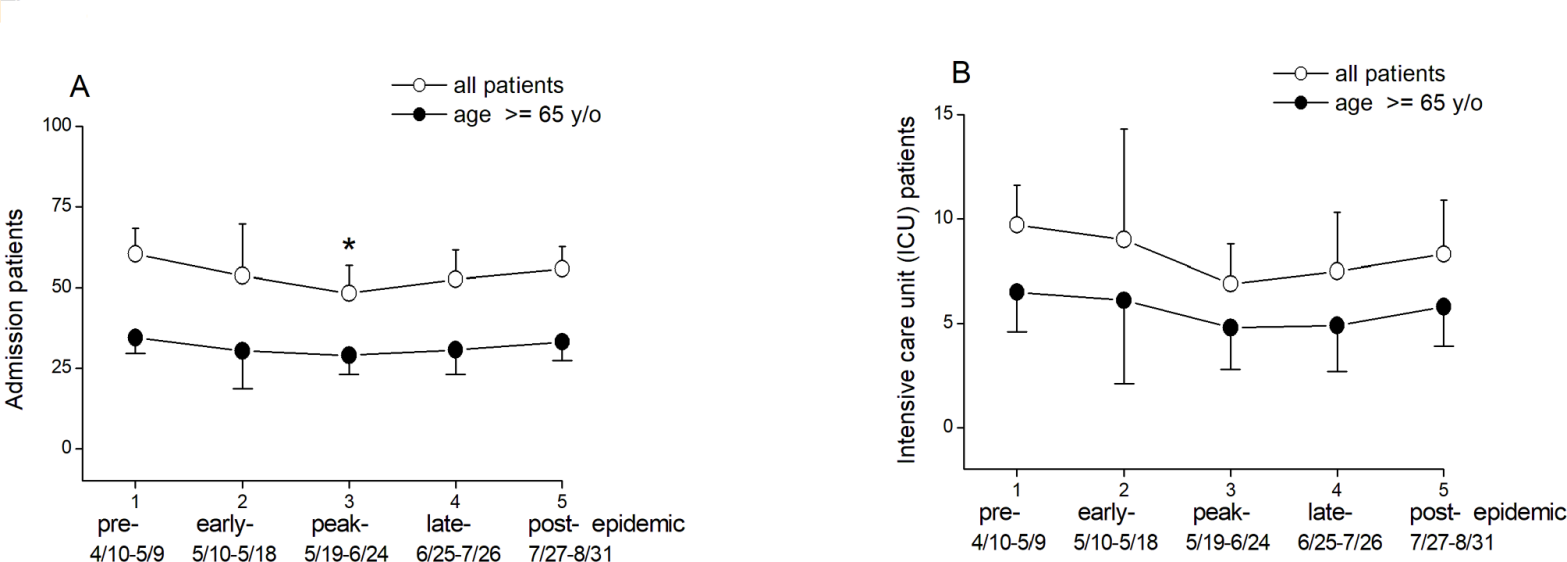
Comparison by admission and intensive care unit category. Comparison between all patients and patients aged ≥ 65 years in admission (**A**) and intensive care unit (ICU) (**B**) category in the pre-epidemic, early-epidemic, peak-epidemic, late-epidemic, and post-epidemic stages during local transmission in Taiwan between April and August 2021. (Values are presented as mean ± standard deviation. *p < 0.0014, compared with the pre-epidemic stage)

Regarding admission (Fig. 4A), the total number of admissions in the general population showed a significantly decrease during the peak- (48.3 ± 8.5, p < 0.001), compared to the pre-epidemic stage (60.3 ± 8.0). But in the older population, the total number of admissions noted no difference during the COVID-19 epidemic.

Regarding ICU hospitalization (Fig. 4B), the total number of ICU admissions in the general population and older population showed no significantly decrease during the COVID-19 epidemic, in both the general (9.7 ± 1.9, 9.0 ± 5.3, 6.9 ± 1.9, 7.5 ± 2.8, and 8.3 ± 2.6) and older population (6.5 ± 1.9, 6.1 ± 4.0, 4.8 ± 2.0, 4.9 ± 2.2, and 5.8 ± 1.9).

## Discussion

As an island country, Taiwan has the advantage of not sharing borders with any other country. This geographical advantage played a significant role in Taiwan’s successful COVID-19 prevention strategy. Unlike many countries worldwide, Taiwan managed to prevent extensive community transmission of the virus, earning praise from numerous countries [10, 18]. Taiwan’s effective handling of the pandemic can be attributed to the lessons learned from the SARS outbreak in 2003. Following the SARS outbreak, Taiwan implemented various measures, such as governmental reorganization, preparedness for medical care systems, and public engagement [22]. These actions provided valuable experience and knowledge that Taiwan utilized in its response to the COVID-19 crisis.

In this study, we demonstrated that the COVID-19 epidemic was associated with a significant reduction in ED visits, affecting both traumatic and non-traumatic patients, which is consistent with findings from a previous study conducted in Thailand [23]. Despite overall the decline in ED visits during the peak phase of the epidemic, we observed that the older patients returned to our ED shortly after the epidemic subsided, in both traumatic and non-traumatic cases. Notably, during the entire period of the epidemic, there was no decrease in the number of admissions among the elderly population, which was different from the general population, suggesting that the older population had an immediate and ongoing need for emergency medical services. Additionally, we observed that the number of patients categorized as triages 1 and 2, indicating a true emergency or an emergency with abnormal vital signs, remained relatively stable throughout all stages of the COVID-19 epidemic. These findings align with a previous study conducted in Italy [24].

The transmission of SARS-associated coronavirus (SARS-CoV) and Middle East respiratory syndrome (MERS) among individuals has been characterized by efficient spread in healthcare facilities, highlighting the vulnerability of our modern healthcare system to nosocomial infection [25]. The aggregation of a large number of sick individuals without proper protection can significantly amplify transmission within hospitals [26]. In contrast to SARS-COV, SARS-COV-2 exhibits high viral shedding in the upper respiratory tract during the early stage of infection. Additionally, a substantial portion of individuals capable of transmitting the virus may be pre-symptomatic, asymptomatic, or only experience mildly symptomatic. These characteristics played a crucial role in facilitating the transmission of COVID-19 primarily within the community during the epidemic. This may help explain the difference in the magnitude of decline between the SARS-CoV and SARS-CoV-2 outbreaks [25].

However, in May 2021, Taiwan faced a concerning situation as it experienced its first significant community spread of COVID-19. There were several domestic cases reported, but only a few could be linked to the initial pilot, while the majority had unknown sources of infection, marking the beginning of community transmission in the country. This outbreak posed the most significant public healthcare crisis that Taiwan experienced since the onset of COVID-19 pandemic. Therefore, understanding the pandemic influence on the ED attendance and utility is important for future health and pandemic planning.

During the first eight months of 2021, when Taiwan experienced its most severe community outbreak, the number of patients visiting our ED was 43,270. This represents an 8.5% reduction compared to the previous year and a marked 25% decrease compared to the number of visits in 2019, just one year before the epidemic was declared. While this decline is already remarkable, it falls short in comparison to the 37–65% reductions reported in studies conducted in other countries [11, 19, 27–30]. In 2002, during the peak of the SARS epidemic in Taiwan, the daily ED visits experienced a reduction of 44–52% compared to the pre-epidemic numbers, which is consistent with the data observed worldwide during the COVID-19 pandemic [31, 32].

The coronavirus pandemic had a significant impact not only on older adults who were infected but also on those who did not have infection. Generally, it is more common for older than younger persons to be characterized by multimorbidity, including hypertension, diabetes mellitus, and malignancy and older persons with these conditions can develop medical complications that require ED visits [33]. Moreover, older adults are at a higher risk of functional decline and may require access to healthcare and supportive services for their chronic conditions. Previous studies have shown that older adults were more likely to cancel or postpone medical appointments during the pandemic [34]. Our study revealed that ED visits by older patients decreased during the peak- and late stages of epidemic but returned to the levels during the post-epidemic stages as pre- and early-epidemic stages. This pattern was not observed in the general population (Fig. 2A). Older patient with chronic respiratory and cardiovascular diseases require close collaboration with healthcare providers and inadequate management of these conditions at home during the lockdown period may put frail older individuals at a higher risk [35]. Furthermore, social isolation and loneliness experienced during lockdown measures had a significant impact on their health [36]. Many individuals deferred seeking medical attention for non-COVID-19 conditions during the pandemic due to concerns about contracting the virus, resulting in reduced visit the ED unless necessary. This poses a significant and often underestimated health risk for geriatric population, including both community-dwelling older adults and nursing home residents [37, 38]. The provision of acute care is crucial for this vulnerable population, particularly during the COVID-19 pandemic. However, it is important to note that not all visits to the ED are true emergencies, and inappropriate utilization to ED service is a well-known issue. This may explain the greater decline in ED visits categorized as triages 3, 4, and 5 during the pandemic, as observed in numerous other studies [39–41].

After successfully flattening the curve and lowering the epidemic alert to Level 2, the total number of patients visiting the ED swiftly increased, but still lower than that in the pre-epidemic stage. However, the number of older patients who sought emergency care for both trauma and non-trauma condition in the post-epidemic stage did not differ significantly from the pre-epidemic stage. To the best of our knowledge, this is the first study demonstrating older patients returning to the ED early when the pandemic slowed down and reached pre-epidemic levels. This observation can be attributed to the higher vulnerability of this patient group to diseases and their urgent need of emergency medical services. Indeed, older adults have concerns about visiting hospitals during the pandemic, but they also prioritize the management of their chronic disease. Factors such as accessibility to comprehensive care, availability, quality of care, and positive past experiences play a crucial role in their decision to seek treatment of non-urgent concerns [42]. In Taiwan, the accessibility and convenience of medical services are well-regarded [43]. Moreover, Taiwan implemented effective control measures to combat the COVID-19 epidemic, which led to quick containment in a few months [44]. These factors likely contribute to the older population’s confidence in seeking medical care and their willingness to return to the ED as the epidemic situation improved. The observed trend of older individuals quickly seeking emergency medical services as the epidemic slowed down can be attributed to factors such as advanced age, higher comorbidity burden, and a greater need for medical care. Older adults are generally more vulnerable and have a higher demand for healthcare services due to their age-related health conditions. In contrast, studies conducted in the UK and US hospitals reported a significant reduction in medical admissions among the oldest age group, possibly due to concerns about acquiring COVID-19 in the hospital or perceived the futility of admission [27, 45]. In the general adult group, there was a gradual increase in ED visits since the late-epidemic stage, but it did not reach the pre-epidemic level once the lockdown measures were lifted. This difference in behavior can be attributed to variations in health behaviors between older and middle-aged adults.

During the peak of the community outbreak in Taiwan, there was a significant increase in the number of newly diagnosed COVID-19 cases on a daily basis. This had a profound impact on the community as the number of infected individuals grew rapidly, posing a risk to public health and straining healthcare resources. At that time, our government also declared a national level 3 epidemic alert, imposing strict lockdown measures and restrictions in an effort to reduce community transmission. These measures included requiring facial coverings in public spaces, banning all social gatherings indoors, and removing all indoor dining by food and beverage vendors. These restrictions were in place to encourage people to stay at home, thus helping to reduce the rate of community transmission with collateral benefits [46]. Our study demonstrated a marked decrease in the number of visiting patients across all groups, including older patient; patients in triage categories 3, 4, or 5; patients visiting for trauma or non-trauma.

However, our study found that the number of patients in triages 1 and 2, representing emergent and urgent conditions, remained unchanged throughout the entire epidemic, which was different from that of some previous studies, which demonstrated a significant decline in the number of older adults for acute unscheduled care [23, 47, 48]. And interestingly, even in the older age group, there was a slight increase in the number of patients since the onset of the epidemic. This discrepancy in findings suggests that the volume of highest-acuity patients, including older adults, either had the smallest reduction or did not decrease during the pandemic, unlike other patient groups [49, 50]. In Saudi Arabia, which implemented a curfew, a more restrictive measure to control virus transmission, patients presenting to the ED during the pandemic were more likely to have a higher level of urgency and acuity [51]. The change in acuity levels of non-COVID-19 ED visits varied across different countries and cities. Emergent and urgent conditions, such as stroke, cardiac problems, and sepsis require immediate attention. In view of stroke, studies have indicated a decline in the volume of admissions for ischemic stroke/TIA admission, overall stroke admission, and mechanical thrombectomy procedures during the COVID-19 pandemic [52, 53]. The reluctance of patients to seek medical attention due to the fear of contracting COVID-19 was considered a possible reason for the decline in admissions for milder stroke cases. Additionally, physical distancing measures may have prevented patients from witnessing stroke symptoms in themselves or others in a timely manner. Acute myocardial infarction was also an emergent condition. Recent studies have reported a significant reduction in admission for ST-elevation myocardial infarction in the United States and Europe during the COVID-19 pandemic [54, 55]. However, in Taiwan, there was no observed reduction in ST-elevation myocardial infarction admissions from February 1 to April 30, 2020 [56]. Theoretically, the actual number of emergent and urgent conditions should remain unchanged during the COVID-19 pandemic. However, the number of patients who had emergent and urgent conditions and visited the ED would be influenced by many factors. In our study, we found that the numbers of patients in triages 1 and 2 remained consistent throughout the entire epidemic. This suggests that the demand for true emergency medical services was resilient and less affected by the magnitude of the COVID-19 community outbreak. These findings highlight the importance of adequate public health strategies and accessible medical care in ensuring that individuals with emergent and urgent conditions receive timely and necessary medical attention during a pandemic.

While the COVID-19 pandemic is beginning to ease, there is no telling whether other infectious diseases may emerge in the future. Our investigation might be useful to understand both the population reaction and healthcare system response at different phases of the pandemic in terms of reduced demand for care and systems capability in intercepting it, especially in geriatrics.

## Limitations

Our study has several limitations. First, it was a retrospective analysis conducted at a single center, which may limit the generalizability of the findings to other healthcare settings or regions. Although our center is the second largest hospital in Taipei, it may not fully represent the experiences of all patients in the country. Second, we only investigated several months during the domestic epidemic and could not predict the effects of a longer COVID-19 epidemic, involving a larger number of patients, on the duration or amplitude of decreases in ED attendance or on further changes in ED disease patterns. Third, the study focused on a COVID-19-dedicated tertiary medical center that primarily serves the geriatric population, which could introduce selection bias and limit the ability to generalize the findings to other healthcare facilities or age groups. The effects of the COVID-19 outbreak on different types of medical centers, non-COVID-19-dedicated medical centers, community hospitals, and private hospitals or clinics or in other cities were not assessed. This could have caused a selection bias. Despite these limitations, the study contributes to our understanding of the impact of COVID-19 on older patients and provides valuable insights into their healthcare utilization during the epidemic.

This study provides invaluable information for the design of new schedules for emergency medical services, workforces of emergency physicians and other specialists, and hospital admission and management systems, which will help medical workers better prepare for any future infectious outbreaks, such as the COVID-19 epidemic. We would also like to emphasize the importance of not overlooking the healthcare needs of the elderly population during the pandemic period, particularly with regard to emergency medical care.

## Conclusion

During the peak of the COVID-19 outbreak, the number of ED visits was significantly affected. However, it is worth noting that, as the epidemic was gradually stabilized, the number of elderly patients is returning to pre-epidemic levels sooner than the general population. Additionally, in the patient group of triage 1&2, which represents a true emergency, the number did not show a drastic change.

## Data Availability

The raw data analyzed during the current study are not publicly available to avoid any possibility of identifying local patients, but aggregated or limited data may be available from the corresponding author on reasonable request.

## List of abbreviations

CECC: Central Epidemic Command Center
COVID-19: coronavirus disease 2019
ED: emergency department
ICU: intensive care unit
SARS-CoV: severe acute respiratory syndrome associated coronavirus
